# The association between cecal insertion time and colorectal neoplasm detection

**DOI:** 10.1186/1471-230X-13-124

**Published:** 2013-08-06

**Authors:** Moon Hee Yang, Juhee Cho, Sanjay Rampal, Eun Kyung Choi, Yoon-Ho Choi, Jun Haeng Lee, Young-Ho Kim, Dong Kyung Chang, Poong-Lyul Rhee, Jae J Kim, Eliseo Guallar, Jong Chul Rhee, Hee Jung Son

**Affiliations:** 1 Center for Health Promotion, Samsung Medical Center, Sungkyunkwan University School of Medicine, Seoul, Korea; 2 Cancer Education Center, Samsung Medical Center, Sungkyunkwan University School of Medicine, Seoul, Korea; 3 Department of Epidemiology, Johns Hopkins University Bloomberg School of Public Health, Baltimore, USA; 4 Julius Centre University of Malaya, Department of Social and Preventive Medicine, Faculty of Medicine, University of Malaya, Kuala Lumpur, Malaysia; 5 Department of Internal Medicine, Samsung Medical Center, Sungkyunkwan University School of Medicine, 50 Irwon-dong, Gangnam-gu, Seoul 135-710, Korea; 6 Department of Medicine and Welch Center for Prevention, Epidemiology, and Clinical Research, Johns Hopkins Medical Institutions, Baltimore, USA

**Keywords:** Cecal insertion time, Colorectal neoplasm, Screening colonoscopy

## Abstract

**Background:**

Information on the impact of cecal insertion time on colorectal neoplasm detection is limited. Our objective was to determine the association between cecal insertion time and colorectal neoplasm detection rate in colonoscopy screening.

**Methods:**

We performed a cross-sectional study of 12,679 consecutive subjects aged 40–79 years undergoing screening colonoscopy in routine health check-ups at the Center for Health Promotion of the Samsung Medical Center from December 2007 to June 2009. Fixed effects logistic regression conditioning on colonoscopist was used to eliminate confounding due to differences in technical ability and other characteristics across colonoscopists.

**Results:**

The mean cecal insertion time was 5.9 (SD, 4.4 minutes). We identified 4,249 (33.5%) participants with colorectal neoplasms, of whom 1,956 had small single adenomas (<5 mm), 595 had medium single adenomas (5–9 mm), and 1,699 had multiple adenomas or advanced colorectal neoplasms. The overall rates of colorectal neoplasm detection by quartiles of cecal insertion time were 36.8%, 33.4%, 32.7%, and 31.0%, respectively (p trend <0.001).The odds for small single colorectal adenoma detection was 16% lower (adjusted OR 0.84; 95% CI 0.71 to 0.99) in the fourth compared to the first quartile of insertion time (p trend 0.005). Insertion time was not associated with the detection rate of single adenomas ≥5 mm, multiple adenomas or advanced colorectal neoplasms.

**Conclusion:**

Shorter insertion times were associated with increased rates of detection of small colorectal adenomas <5 mm. Cecal insertion time may be clinically relevant as missed small colorectal adenomas may progress to more advanced lesions.

## Background

Early detection and removal of colorectal adenomas by colonoscopy decreases the risk of colorectal cancer [1], and colonoscopy has become the standard for detection of colorectal neoplasms. However, the miss rate for colonoscopy is not negligible and up to 5% of new colorectal cancer cases had a colonoscopy in the previous 3–5 years [2-6]. Indeed, the adenoma detection rate is an independent predictor of the risk of interval colorectal cancer after screening colonoscopy [7], and measuring the adenoma detection rates of individual colonoscopists is a priority in quality improvement in colonosocopy units [8].

Higher adenoma detection rates are associated with more careful examination of the proximal sides of folds and flexures, with adequate distension, with cleaning of pools of fluid and dirty areas, and with time for scope withdrawal [9,10]. Taking adequate time during withdrawal is necessary for meticulous mucosal examination and several studies have demonstrated that colonoscopy withdrawal time is a major determinant of adenoma miss rates [10-13]. Shorter cecal insertion time in relation to withdrawal time was associated with higher adenoma detection rates in a previous small study [14]. However, more research is needed to determine the appropriateness of using insertion time as a quality indicator for colonoscopy. The aim of this study was to investigate the association between cecal insertion time and colorectal neoplasm detection in a large colonoscopy screening clinic.

## Methods

### Study population

We conducted a retrospective study of screening colonoscopies at the Center for Health Promotion of the Samsung Medical Center in Seoul, Korea, from December 2007 to June 2009. We restricted the study to male and female participants aged 40 to 79 years old as this is the recommended age range for screening colonoscopy in Korea. From a total of 14,370 consecutive colonoscopies performed at the Center for Health Promotion, we excluded 862 colonoscopies conducted in participants who were <40 or ≥80 years of age, 526 colonoscopies performed for therapeutic (non-screening) purposes, 65 colonoscopies performed outside of the colonoscopy unit, 29 colonoscopies performed in participants with a history of colorectal cancer, inflammatory bowel disease or who had undergone colonic resection, and 113 colonoscopies with incomplete examination due to poor bowel preparation. In subjects who had colonoscopy multiple times during the study period (n = 98), we chose the first colonoscopy results. Because some individuals met more than one exclusion criterion, the final sample size was 12,679 (7,975 men and 4,704 women). The study protocol was reviewed and approved by the institutional review board at Samsung Medical Center. The informed consent requirement was exempted by the Institutional Review Board because researchers only accessed retrospectively a de-identified database for analysis purposes.

### Study procedures

Twenty three board-certified gastroenterologists performed the colonoscopies. Each colonoscopist had performed more than 500 examinations before the study period or before working at the Center for Health Promotion. Colonoscopies were performed after bowel preparation with 4 L polyethylene glycol solution (Colyte®, Taejun, Seoul, Korea; Colyte®-F, Taejun, Seoul, Korea; Colonlyte®, Dreampharma, Seoul, Korea).

Cecal insertion time was defined as the time from insertion into the rectum to the time when the colonoscope tip passed to a point proximal to the ileocecal valve so that the base of cecum was visible. Withdrawal time was defined as the time taken for withdrawing the colonoscope tip from the base of cecum to across the anus. Cecal insertion and withdrawal times were recorded immediately after finishing the examination by the colonoscopist. Bowel preparation was assessed as excellent (no or nearly no fecal matter in the colon; small-to-moderate amounts of clear liquid present), good (small amounts of thin liquid fecal matter visible and easily suctioned, mainly distal to the splenic flexure), fair (moderate amounts of thick liquid to semisolid fecal matter visible and suctioned, including proximal to the splenic flexure; >90% of the mucosa visible), or poor (large amounts of solid fecal matter present that preclude a satisfactory study; <90% of the mucosa visible).

Colorectal neoplasms were further classified as small single non-advanced adenomas < 5 mm, medium single non-advanced adenomas 5 to <10 mm,and multiple or advanced colorectal neoplasms (including multiple adenomas regardless of size and advanced colorectal neoplasms). Advanced adenoma was defined as a tubular adenoma with diameter ≥10 mm, an adenoma with villous component, or an adenoma with high grade dysplasia. The size of each lesion was estimated using open biopsy forceps.

A health questionnaire and a detailed physical exam were routinely completed as part of the screening program. Height and weight were measured using an Inbody 720 machine (Biospace, Seoul, Korea). Body mass index (BMI) was calculated by dividing measured weight (kg) by height squared (m^2^). Waist circumference was measured at the midpoint between the inferior margin of the last rib and the superior iliac crest in a horizontal plane. Information on history of colorectal polyps, diabetes mellitus, hyperlipidemia, medication use including aspirin and non-steroidal anti-inflammatory drugs (NSAIDs), alcohol drinking and smoking and family history of colorectal cancer were collected using a self-administered questionnaire before endoscopy.

### Statistical analysis

Colorectal adenoma proportions were compared across the quartiles of cecal insertion time. Odds ratios (ORs) and 95% confidence intervals (CIs) for the presence of any colorectal neoplasm for the 3 highest quartiles of insertion time compared to the first quartile were calculated using fixed effects logistic regression conditioned on colonoscopists. These regression models account for data clustering by colonoscopists and eliminate confounding due to differences across colonoscopists. Separate logistic regression models were used for the different colorectal neoplasms. In multivariable models, we adjusted for age (40 to 49, 50 to 59, 60 to 69, 70 to 79 years), sex, withdrawal time (<6 minutes, ≥6 minutes), bowel preparation (excellent, good, fair and poor), BMI (<25, ≥25 kg/m^2^), waist circumference (<80, 80 to 89, 90 to 99, ≥100 cm), family history of colorectal cancer, history of colorectal polyps, diabetes, hyperlipidemia, aspirin use, NSAIDs use, calcium use, alcohol drinking, and smoking (current, past, and never).

Responses to health questionnaire items coded as “unknown” and items not answered were considered as missing data. We used multiple imputations with chained equations to address missing data [15]. A total of 20 imputed sets were created, each a result of 1000 iterations. Missing covariate patterns were individually explored and imputation equations refined. The residuals of the imputation regression models were graphically explored as a form of diagnostics. All analysis on imputed data accounted for the imputed nature of the datasets. Two sided p-values < 0.05 were considered statistically significant. Statistical analyses were performed using Stata version 12.0 (Stata Corp, Texas, USA).

## Results

The mean (SD) age of study participants was 53.4(7.3) years and 62.9% of study participants were male (Table 1). The proportions of participants with a family history of colorectal cancer and with a personal history of colorectal polyps were 3.6% and 18.1%, respectively. The mean (SD) cecal insertion time was 5.9 (4.4) minutes and the mean (SD) withdrawal time was 9.0 (3.9) minutes.

**Table 1:** Characteristics of study participants (N = 12,679)

**Characteristic**	**N available**	**% missing**	**Number (%) or mean ± SD**
Age, years	12,679	0.0	53.4 ± 7.3
40-49			4,215 (33.2)
50-59			5,882 (46.4)
60-69			2,296 (18.1)
70-79			286 (2.3)
Male sex	12,679	0.0	7,975 (62.9)
Body mass index, kg/m^2^	12,091	4.6	24.2 ± 2.8
Waist circumference, cm	10,803	14.8	84.1 ± 8.8
Family history of colorectal cancer	12,679	0.0	458 (3.6)
History of colorectal polyp	11,188	11.8	2,030 (18.1)
Diabetes mellitus	11,237	11.4	975 (8.7)
Hyperlipidemia	11,189	11.8	2,155 (19.3)
Aspirin use	10,878	14.2	1,529 (14.1)
NSAIDs use	10,878	14.2	321 (3.0)
Calcium use	10,917	13.9	1,033 (9.5)
Currently consumes alcohol	11,144	12.1	7,142 (64.1)
Current smoker	11,406	10.0	2,150 (18.8)
Quality indicators of colonoscopy			
Insertion time (minutes)	12,260	3.3	5.9 ± 4.4
Withdrawal time (minutes)	12,239	3.5	9.0 ± 3.9
Excellent/Good Bowel preparation	11,805	6.9	8,086 (68.5)

Their median age of the 23 colonoscopists was 34 years (interquartile range, 32 to 36). A majority of the colonoscopists were female (82.6%). The median number of colonoscopies performed by each colonoscopist during the study period was 592 (interquartile range, 416 to 709). Before the study period, the 23 colonoscopists had performed a median of 1,400 colonoscopies (interquartile range, 1,200 to 1,900).

Colorectal neoplasms were detected in 4,249 participants (33.5%; Table 2). The adenoma detection rates ranged from 23.7% to 48.3% across colonoscopists. The proportion of overall colorectal neoplasm detection by quartiles of cecal insertion time were 36.8%, 33.4%, 32.7%, and 31.0% (p trend <0.001). The detection rate of small single colorectal adenoma decreased with higher quartiles of insertion time: 17.7%, 15.1%, 14.9%, and 13.9% (p trend <0.001). The detection rate of multiple adenomas or advanced colorectal neoplasms also decreased with higher quartiles of insertion time: 14.4%, 13.6%, 13.0%, and 12.5% (P trend 0.019). There was no evidence of an association between insertion time and the detection rates of single adenomas 5–9 mm.

**Table 2:** Colorectal neoplasm detection by quartile of colonoscopy cecal insertion time (N = 12,679)

	**N (%)**	**Quartiles of cecal insertion time, % (SE%)**				
		**First**	**Second**	**Third**	**Fourth**	**P value**
		**(<3.1 min)**	**(3.1-4.6 min)**	**(4.7-7.1 min)**	**(≥7.2 min)**	
No colorectal lesion	8,429 (66.5)	63.2 (0.8)	66.6 (0.9)	67.3 (0.8)	69.0 (0.8)	
Any colorectal lesion detection	4,250 (33.5)	36.8 (0.8)	33.4 (0.9)	32.7 (0.8)	31.0 (0.8)	<0.001
Small single adenoma, <5 mm	1,956 (15.4)	17.7 (0.7)	15.1 (0.7)	14.9 (0.6)	13.9 (0.6)	<0.001
Medium single adenoma, 5-9 mm	595 (4.7)	4.7 (0.4)	4.7 (0.4)	4.8 (0.4)	4.6 (0.4)	0.86
Multiple adenomas or advanced colorectal neoplasm	1,699 (13.4)	14.4 (0.6)	13.6 (0.6)	13.0 (0.6)	12.5 (0.6)	0.019

**Figure 1:**
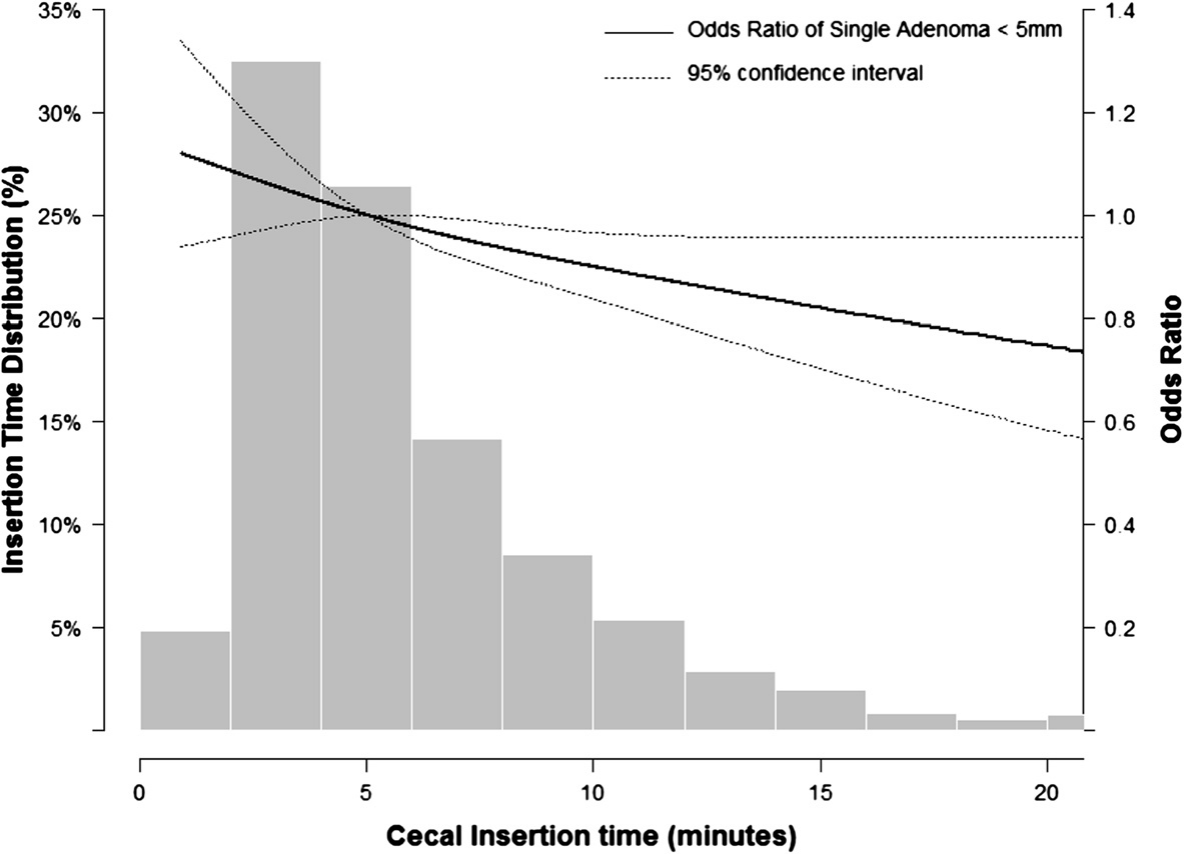
**Prevalent odds ratio of single colorectal adenoma < 5 mm.** Adjusted relative prevalence odds ratios derived from spline logistic regression models using restricted cubic splines with knots at the 10th, 50th, and 90th percentiles of the distribution of the insertion time distribution. The reference value (odds ratio = 1) was set at the 5th minute of insertion time. Prevalent odds ratios were adjusted for age, sex, body mass index, waist circumference, family history of colorectal cancer, history of colorectal polyp, diabetes mellitus, hyperlipidemia, aspirin medication, other NSAID medication, calcium supplementation, alcohol use, smoking history, colonoscopist, and bowel preparation (see Table 3 for details).

The inverse association between insertion time and small single colorectal adenoma persisted in multivariable analyses (Table 3). The odds for small single colorectal adenoma detection was 16% lower (adjusted OR 0.84; 95% CI 0.71 to 0.99) in the fourth compared to the first quartile of insertion time (p trend 0.005). Sensitivity analyses using complete case analyses (N = 12,260) instead of multiple imputation for missing data showed similar results (Additional file 1: Table S1 and Additional file 2: Table S2). In analyses using insertion time as a continuous variable, the multivariable adjusted odds ratios (95% CI) for colorectal neoplasm detection associated with a 5 minute increase in insertion time were 0.90 (95% CI 0.84 to 0.97) for single adenomas < 5 mm, 1.05 (0.94 to 1.16) for single adenomas 5 to 9 mm and 1.00 (0.93 to 1.07) for multiple adenomas or advanced colorectal neoplasms. In multivariable restricted cubic spline models, shorter cecal insertion times were also associated with higher detection rates of small <5 mm single colorectal adenomas (Figure 1).

**Table 3:** Odds ratios for colorectal neoplasm detection by colonoscopy cecal insertion times (N = 12,679)

	**Quartiles of cecal insertion time, Odds Ratio (95% CI)**					
	**First**	**Second**	**Third**	**Fourth**	**Per 5-minute increase**	**P-trend**
	**(<3.1 min)**	**(3.1-4.6 min)**	**(4.7-7.1 min)**	**(≥7.2 min)**		
**Any colorectal neoplasms**						
Crude OR* (95% CI)	1.00 (ref.)	0.87 (0.78, 0.97)	0.93 (0.83, 1.04)	0.90 (0.80, 1.02)	0.97 (0.92, 1.01)	0.16
Adjusted OR** (95% CI)	1.00 (ref.)	0.95 (0.85, 1.06)	1.01 (0.89, 1.13)	0.94 (0.83, 1.07)	0.96 (0.91, 1.01)	0.13
**Small single adenoma, < 5 mm**						
Crude OR* (95% CI)	1.00 (ref.)	0.80 (0.69, 0.92)	0.83 (0.72, 0.96)	0.77 (0.66, 0.91)	0.89 (0.83, 0.95)	0.001
Adjusted OR** (95% CI)	1.00 (ref.)	0.87 (0.75, 1.00)	0.91 (0.78, 1.06)	0.84 (0.71, 0.99)	0.90 (0.84, 0.97)	0.005
**Medium single adenoma, 5-9 mm**						
Crude OR* (95% CI)	1.00 (ref.)	1.00 (0.78, 1.28)	1.10 (0.86, 1.41)	1.08 (0.83, 1.41)	1.05 (0.94, 1.16)	0.37
Adjusted OR** (95% CI)	1.00 (ref.)	1.05 (0.82, 1.35)	1.16 (0.89, 1.50)	1.11 (0.84, 1.46)	1.05 (0.94, 1.16)	0.40
**Multiple adenomas or advanced colorectal neoplasm**						
Crude OR* (95% CI)	1.00 (ref.)	0.93 (0.80, 1.08)	0.99 (0.85, 1.16)	1.02 (0.86, 1.20)	1.02 (0.96, 1.09)	0.52
Adjusted OR** (95% CI)	1.00 (ref.)	1.03 (0.88, 1.22)	1.09 (0.92, 1.29)	1.04 (0.87, 1.24)	1.00 (0.93, 1.07)	0.99

* Crude OR conditions on colonoscopist.

** Adjusted OR adjusts for age, sex, body mass index, waist circumference, family history of colorectal cancer, history of colorectal polyp, diabetes mellitus, hyperlipidemia, aspirin medication, other NSAID medication, calcium supplementation, alcohol use, smoking history, colonoscopist, and bowel preparation.

## Discussion and conclusions

In this large study of routine screening colonoscopy practice, shorter cecal insertion times were associated with higher detection rates of adenomas, although the association was restricted to small adenomas. This association persisted after controlling for colonoscopist and after adjusting for a variety of patient and colonoscopy characteristics, including withdrawal time and bowel preparation. Since small adenomas comprise a large proportion of adenomas and missing them may increase the likelihood of colorectal cancers, our findings may be relevant from a clinical perspective.

Cecal insertion time is determined by multiple colonoscopist- and patient-related factors. Insertion time may reflect the colonoscopist’s skill, concentration, or fatigue. Experienced colonoscopists inserting the scope to the cecum without forming colonic loops are likely to have shorter insertion times. Examining the colon after straightening allows for a more controlled pull back, with easier reinsertion to recheck difficult and potential blind areas [16]. Withdrawal through a large loop complicates control of the colonoscope and may result in skipping several inches of the colon without having been inspected at all [16]. A good insertion technique is thus essential for a good examination of the colonic mucosa. A fast and efficient insertion technique may also make it easier for the colonoscopist to keep his/her concentration during withdrawal. Insertion time may also be a marker for overall proficiency of the colonoscopist, as lower annual case volumes are associated with prolonged insertion times [17]. We note, however, that our analyses conditioned on colonoscopist, so that variations in ability and other differences across colonoscopists cannot explain the inverse association that we observed between insertion time and small adenoma detection rate.

Prolonged insertion times also depend on patient-related factors and may reflect a more difficult examination [18]. Prolonged cecal insertion time can be due to a redundant and tortuous colon, which may likely require prolonged withdrawal for a thorough inspection of the mucosa [14]. Older age, female sex, poor bowel preparation, smaller waist circumference, lower BMI, history of prior abdominal hysterectomy, and constipation have all been associated with increased cecal insertion times [17-20]. In our study, however, adjusting for a variety of procedure- and patient-related factors, including withdrawal time, did not materially affect the observed inverse association between insertion time and small adenoma detection. In a prior study, colonoscopists with mean cecal insertion to mean withdrawal time ratios <1 detected significantly more adenomas compared to colonoscopists with ratios >1 [14]. Stress from longer insertion times and scheduling pressure may influence the observed cecal intubation to withdrawal time relationship [14]. The higher miss rate in patients with longer cecal insertion times may thus be related to patient or colonic related factors, to endoscopists’ fatigue from a difficult colonoscopy, or to stress from scheduling. Therefore, a longer insertion time may increase the likelihood that an endoscopist misses smaller adenomas in relatively difficult to visualize areas of the colon. We similarly found that cecal insertion time was negatively related to withdrawal time (results not shown). In our study, we used the insertion times of each colonoscopy instead of the mean insertion times of each colonoscopist and we controlled for colonoscopist to exclude that the observed association was due to other differences across colonoscopists. In another large study of primary care physician-performed colonoscopies with standby specialist support, mean insertion time was significantly longer in cases with polyps [21]. However, colonoscopies performed by primary care physicians with lower colonoscopy volumes and mean insertion times were much longer than those of experienced board-certified gastroenterologists in other studies. A recent small study reported that insertion times > 5 min and withdrawal time <10 min were associated with a higher miss rate [22], but this study was restricted to patients who underwent a second colonoscopy for polypectomy and its findings are not directly applicable to all patients undergoing screening colonoscopy.

While the clinical implications and optimal management of small polyps are controversial [23,24], several lines of evidence indicate that detection of small polyps may result in long-term benefits for patients [12,25]. In a close follow-up of 30 polyps during 2 years, the mean tubular adenoma growth rate was 0.58 mm/year and 3 tubular adenomas had growth rates >2 mm/year [26]. No polyp regressed completely [26]. There is evidence that although the presence of cancer at the time of detection is uncommon, small adenomas may eventually develop into cancer [27]. Furthermore, small adenomas can be easily removed by hot or cold biopsy forceps, with lower risk of bleeding compared to polypectomy [27].

Our study had several strengths, including the large sample size, the use of unselected participants undergoing routine screening colonoscopy, and the availability of detailed covariate information which allowed us to consider in the analysis many patient- and procedure-related factors that affect the prevalence and detection rates of colorectal neoplasms. Also the use of fixed effects regression conditional on colonoscopist also eliminates confounding by potential differences across colonoscopists.

Several limitations to our study, however, also need to be considered in the interpretation of our findings. First, we could not determine whether polyps were detected or removed during insertion or withdrawal of the colonoscope. Removal of diminutive polyps during insertion, however, cannot explain an inverse association between shorter insertion times and adenoma detection rate. Second, we did not have data on the use of sedative or antispasmodic drugs during colonoscopy, which may influence cecal insertion time and adenoma detection rate. Third, our questionnaire captured history of colorectal polyps but not of colorectal adenomas, and some participants could not remember the results of previous colonoscopies. However, our findings were similar in the subgroup of study participants who reported a negative history of colorectal polyps. Fourth, we did not have information on compliance to preparation procedures. We could not assess the consistency of colonoscopists to adjudicate bowel preparation score criteria that may be subject to intra- and inter-observer variation. Fifth, we could not investigate practice-related factors, including number of colonoscopies performed per day and daily total procedure time that may influence colonoscopist performance. Sixth, polyp sizes were measured by eye using open biopsy forceps. Therefore, exact measurements of polyp size were not available and intra- and inter-observer variation may add to random variability of analyses by tumor size. Finally, as our study included endoscopists and patients from a single center, generalizability of these findings to other populations is uncertain.

In conclusion, we found an inverse association between cecal insertion time and the detection of small colorectal adenomas. Since missed colorectal adenomas may progress to more advanced lesions, our findings may be relevant from a clinical perspective. In our analysis, we controlled for the effects of individual endoscopists. As a consequence, our data suggest that endoscopists should pay close attention to the detection of adenomas in cases that need longer insertion times.

## Competing interest

The authors declare that they have no competing interest.

## Authors’ contributions

Conception and design: MHY, JC, JHL, HJS, EG. Acquisition of data: MHY, EKC, Y-HC, JHL, Y-HK, DKC, P-LR, JJK, JCR. Analysis and interpretation of data: MHY, JC, SR, EG, HJS. Manuscript writing: MHY, JC, SR, EG. Critical revision of manuscript: EG, JHL, Y-HK, DKC, P-LR, JJK, JCR, Y-HC, EKC, HJS. Study supervision: EG, HJS. Final approval of manuscript: EG, HJS, MHY, JC, SR, EKC, Y-HC, JHL, Y-HK, DKC, P-LR, JJK, JCR. All authors read and approved the final manuscript.

## Pre-publication history

The pre-publication history for this paper can be accessed here:

http://www.biomedcentral.com/1471-230X/13/124/prepub

## Supplementary Material

Additional file 1: Table S1 Colorectal neoplasm detection by quartile of cecal colonoscopy insertion time (complete case analysis, N = 12,260). Click here for file

Additional file 2: Table S2 Odds ratios for colorectal neoplasm detection by cecal colonoscopy insertion times (Complete case analysis). Click here for file
