# Supplementary material for: The association between cecal insertion time and colorectal neoplasm detection
Source: BMC Gastroenterol. 2013 Aug 6;13:124. doi: 10.1186/1471-230X-13-124 (PMC3750659; doi:10.1186/1471-230X-13-124)
Supplement: Additional file 1: Table S1 — Colorectal neoplasm detection by quartile of cecal colonoscopy insertion time (complete case analysis, N = 12,260). [file 1471-230X-13-124-S1.doc]

**Table S1. Colorectal neoplasm detection by quartile of cecal colonoscopy insertion time (complete case analysis, N = 12,260).**

|  | Quartiles of cecal insertion time, % (SE%) | | | | |
| --- | --- | --- | --- | --- | --- |
|  | First (<3.1 min),  N=3,243 | Second (3.1-4.6 min),  N=2,923 | Third (4.7-7.1 min),  N=3,072 | Fourth (≥7.2 min),  N=3,022 | P value |
| No colorectal lesion | 63.2 (0.8) | 66.7 (0.9) | 67.5 (0.8) | 69.2 (0.8) |  |
| Any colorectal lesion detection | 36.8 (0.8) | 33.3 (0.9) | 32.5 (0.8) | 30.8 (0.8) | <0.001 |
| Small single adenoma, <5mm | 17.9 (0.7) | 15.1 (0.7) | 14.9 (0.6) | 13.9 (0.6) | <0.001 |
| Medium single adenoma, 5-9mm | 4.7 (0.4) | 4.7 (0.4) | 4.8 (0.4) | 4.6 (0.4) | 0.45 |
| Multiple adenomas or  advanced colorectal neoplasm | 14.2 (0.6) | 13.5 (0.6) | 12.8 (0.6) | 12.3 (0.6) | 0.001 |
