# Supplementary material for: The association between cecal insertion time and colorectal neoplasm detection
Source: BMC Gastroenterol. 2013 Aug 6;13:124. doi: 10.1186/1471-230X-13-124 (PMC3750659; doi:10.1186/1471-230X-13-124)
Supplement: Additional file 2: Table S2 — Odds ratios for colorectal neoplasm detection by cecal colonoscopy insertion times (Complete case analysis). [file 1471-230X-13-124-S2.doc]

**Table S**2: Odds ratios for colorectal neoplasm detection by cecal colonoscopy insertion times (Complete case analysis)

|  | Quartiles of cecal insertion time, Odds Ratio (95% CI) | | | | | |
| --- | --- | --- | --- | --- | --- | --- |
|  | First (<3.1 min) | Second (3.1-4.6 min) | Third (4.7-7.1 min) | Fourth (≥7.2 min) | Per 5-minute increase | P-trend |
| **Any colorectal neoplasms** |  |  |  |  |  |  |
| Crude OR (95% CI) | 1.00 (ref.) | 0.87 (0.78, 0.97) | 0.93 (0.83, 1.04) | 0.91 (0.81, 1.02) | 0.97 (0.92, 1.01) | 0.166 |
| Adjusted OR (95% CI) | 1.00 (ref.) | 1.06 (0.92, 1.22) | 1.11 (0.96, 1.29) | 1.00 (0.86, 1.17) | 0.96 (0.91, 1.03) | 0.258 |
| **Small single adenoma, < 5mm** |  |  |  |  |  |  |
| Crude OR (95% CI) | 1.00 (ref.) | 0.79 (0.68, 0.91) | 0.83 (0.71, 0.96) | 0.77 (0.66, 0.91) | 0.89 (0.83, 0.95) | 0.001 |
| Adjusted OR (95% CI) | 1.00 (ref.) | 0.92 (0.77, 1.11) | 0.98 (0.81, 1.18) | 0.89 (0.73, 1.09) | 0.90 (0.83, 0.98) | 0.021 |
| **Medium single adenoma, 5-9mm** |  |  |  |  |  |  |
| Crude OR (95% CI) | 1.00 (ref.) | 1.01 (0.79, 1.29) | 1.11 (0.86, 1.42) | 1.09 (0.84, 1.42) | 1.05 (0.95, 1.17) | 0.347 |
| Adjusted OR (95% CI) | 1.00 (ref.) | 1.20 (0.88, 1.63) | 1.26 (0.91, 1.74) | 1.18 (0.84, 1.67) | 1.05 (0.92, 1.19) | 0.470 |
| **Multiple adenomas or**  **advanced colorectal neoplasm** |  |  |  |  |  |  |
| Crude OR (95% CI) | 1.00 (ref.) | 0.93 (0.80, 1.09) | 0.99 (0.85, 1.16) | 1.02 (0.86, 1.21) | 1.02 (0.96, 1.09) | 0.514 |
| Adjusted OR (95% CI) | 1.00 (ref.) | 1.25 (1.02, 1.53) | 1.32 (1.06, 1.63) | 1.14 (0.90, 1.43) | 1.01 (0.93, 1.10) | 0.785 |

* Crude OR conditions on colonoscopist. Multivariate (adjusted) OR adjusts for age, sex, body mass index, waist circumference, family history of colorectal cancer, history of colorectal polyp, diabetes mellitus, hyperlipidemia, aspirin medication, other NSAID medication, calcium supplementation, alcohol use, smoking history, colonoscopist, and bowel preparation. Complete case fully adjusted model has a sample size of 7,847.
